# Polymer Composites with Cork Particles Functionalized by Surface Polymerization for Fused Deposition Modeling

**DOI:** 10.1021/acsapm.1c01632

**Published:** 2022-01-13

**Authors:** Alberto S. de León, Fernando Núñez-Gálvez, Daniel Moreno-Sánchez, Natalia Fernández-Delgado, Sergio I. Molina

**Affiliations:** Dpto. Ciencia de los Materiales, I. M. y Q. I., IMEYMAT, Facultad de Ciencias, Universidad de Cádiz, Campus Río San Pedro, s/n, Puerto Real (Cádiz) 11510, Spain

**Keywords:** cork, composites, additive manufacturing, fused deposition modeling, surface modification, circular economy, renewable resources

## Abstract

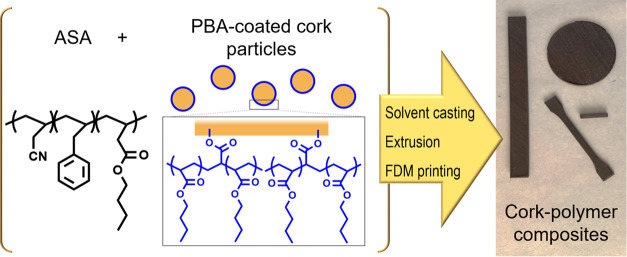

Cork powder received as a byproduct from local industries is valorized through the development of composite materials suitable for fused deposition modeling (FDM). For this purpose, a polymeric matrix of acrylonitrile–styrene–butyl acrylate (ASA) is used due to its good mechanical resistance and weather resistance properties. Prior to the manufacturing of the composites, the cork particles are characterized and modified by surface polymerization, creating a layer of poly(butyl acrylate) (PBA). Then, filaments for FDM are prepared by solvent casting and extrusion from ASA and composites with unmodified cork (ASA + C) and PBA-modified cork (ASA + C_m_). PBA is one of the polymers present in the structure of ASA, which increases the compatibility between the cork particles and the polymer matrix. This is evidenced by evaluating the mechanical properties of the composites and examining their fracture surface by scanning electron microscopy. The analysis of the thermal properties shows that the developed composites also present enhanced insulating properties.

## Introduction

The production of new sustainable materials is one promising trend that has caught the attention of science and industry due to the many environmental benefits and the use of resources or even waste at low cost. In this regard, in the frame of circular economy, composite materials fabricated from waste or byproducts from other industries suppose an important strategy of valorization, which allow the creation of new materials with new structural or functional properties.^1−3^ These strategies include the use of renewable resources as feedstock for the production of polymer-based composites, which allows the successfully processing of new materials for different technologies at the industrial scale.^4^

Cork is a renewable and sustainable raw material that can be extracted from the bark of oaks that naturally grow in Mediterranean regions such as Italy, Spain, or Portugal. Cork has been used for many centuries in different applications due to its unique properties such as extremely low density, high acoustic and thermal insulation behavior, high friction coefficient, high resilience and a Poisson coefficient of virtually 0.^5−7^ For instance, cork is used in sandwich structures as a structural material to minimize the weight of panels and provide good insulating properties. These panels can tolerate loads with a high impact, competing even with glass fibers, being a more sustainable alternative, which makes them attractive in many different applications ranging from civil to aerospace engineering, the automotive sector, or defense. Its acoustic and anti-vibration properties also make it attractive for the design of cabins for planes or turbines or joints in submarines.^8−10^

However, the cork powder generated at the industrial scale is considered as a residue, and it is generally burnt or disposed in landfills. During these processes, up to 30 wt % of the raw material is converted into powder, which may result in a good opportunity to develop new products.^11^ For instance, cork powder can be added to concrete in the construction sector to provide thermal and acoustic insulation, while it contributes to the creation of a more lightweight material, which has enormous consequences in terms of energy saving.^12,13^

Cork powder-based composites using a polymer matrix have also been developed in order to obtain more environmentally friendly materials. There are different studies with polyolefins such as polyethylene (PE) or polypropylene (PP),^14,15^ as well as with biodegradable polymeric matrixes such as acid polylactic acid (PLA),^16^ polycaprolactone (PCL),^17^ or poly(3-hydroxybutyrate-*co*-3-hydroxyvalerate) (PHBV).^18^ Most of these composites are processed via classical manufacturing techniques, typically by pultrusion, injection, or compression molding. However, there is an increasing interest in the development of composite materials suitable for additive manufacturing.^19^

Among the different additive manufacturing techniques, fused deposition modeling (FDM) is the most widespread technique employed for polymeric materials and polymer-based composites. Briefly, in this technology, a polymeric material in the form of a filament or pellets is extruded through a preheated nozzle and is deposited on a platform layer by layer.^20^ The most used thermoplastic polymers are PLA and poly(acrylonitrile–butadiene–styrene) (ABS). PLA is vastly used for domestic applications or sectors that do not require working outdoors or at temperatures above 60 °C. This is why for most of the engineering applications, ABS is preferred since it has similar mechanical properties to PLA but can tolerate working temperatures up to 100 °C.^21^ In the last few years, poly(acrylonitrile–styrene–butyl acrylate) (ASA) has come up as an interesting alternative to ABS. Replacing the butadiene residues with butyl acrylate gives ASA greater environmental resistance.^22^

Recent advances have reported the use of biodegradable materials such as flax, coffee beans or tree bark as reinforcing materials for filaments suitable for FDM.^23^ However, if these composites are not adequately compounded, they may present heterogeneities, which lead to cracks and delamination, causing the embrittlement of the material.^24,25^ In the case of cork particles, their shape, size and microstructure have a great effect on the rheology of the material during the processing and on the mechanical properties of the manufactured composites.^26^ Moreover, the modification of its wetting properties is also critical to enhance the interfacial adhesion with a hydrophobic polymer matrix. The strategies adopted to overcome these drawbacks include adding plasticizers, copolymers or even lignin and suberin during the compounding,^15,27^ as well as the chemical modification of either the polymer matrix or the cork to chemically modify their surface properties.^28^ For instance, previous reports indicate an enhancement of the adhesion properties for PLA-based composites when maleic anhydride is grafted onto the surface of PLA^26^ or the cork particles surface is acetylated, increasing their hydrophobicity.^29^ Other authors modified the surface of the cork particles through a controlled polymerization reaction via atom-transfer radical polymerization. For this purpose, the cork particles were first brominated to be able to perform in a second step the polymerization of methyl methacrylate (MMA) selectively in their surface following a “grafting from” approach. This conferred the cork particles the same surface chemistry as the PMMA matrix used in the composite, effectively enhancing their compatibility.^30^

In this paper, a series of cork-based composites suitable for 3D printing by FDM are developed. Taking into account the low compatibility of cork with the ASA polymer matrix, due to their different surface chemistries, a modification of the cork particles is explored via surface polymerization of poly(butyl acrylate) (PBA) before manufacturing the composites. Then, filaments valid for FDM are prepared by solvent casting and extrusion, and different specimens are printed. The cork-based composites exhibited higher strength and stiffness after surface modification with PBA, compared to composites prepared with cork as received. Furthermore, these composites showed significantly lower thermal conductivities than ASA, demonstrating that they are good candidates for lightweight, insulating materials that can be mass-produced by additive manufacturing technologies.

## Materials and Methods

### Materials

Cork particles with a diameter of 63–125 μm and an average density of ρ = 200 kg/m^3^ were supplied by the company Corchos del Estrecho, obtained from cork dust residues from the sanding processes during the manufacture of cork stoppers. ASA pellets (ASA LI912, ρ = 1100 kg/m^3^) were purchased from LG Chem. Acetic anhydride, pyridine, and sodium hydroxide were purchased from Sigma-Aldrich. Acryloyl chloride (96%), triethylamine (TEA), *n*-butyl acrylate (*n*BA, +98%) and 2,2′-azobis(2-methylpropionitrile) (AIBN) were purchased from Alfa Aesar. Toluene, dimethylformamide (DMF), dichloromethane (DCM), and isopropanol were purchased from Scharlau.

### Hydroxyl Number of Cork Particles

The hydroxyl number (OHN) is defined as the amount of available −OH groups on the cork surface per gram of cork. It was calculated by adapting the ASTM D1957 standard to estimate the OHN of fatty acids. In our case, 15 mL of acetic anhydride and 110 mL of pyridine were used per 1 g of cork particles (C). The mixture was added to a round flask under an inert atmosphere at 100 °C for 2 h. Then, 10 mL of distilled water was added to hydrolyze the non-reactant anhydride, and the amount of acetic acid produced was titrated using a 0.5 M NaOH solution and phenolphthalein as an indicator. This was done three times to ensure the reproducibility of the results. For each repeat, a control in the absence of cork particles was done. The exact details of the procedure are described in the Supporting Information.

### Synthesis of PBA-Modified Cork (C_m_)

The synthesis of C_m_ was carried out in two steps. First, 3.72 g of C was dispersed in 241 mL of DMF and 6.93 mL of TEA by magnetic stirring in a round flask under an inert atmosphere at 0 °C. Then, 2.12 mL of acryloyl chloride was added dropwise. After 3 h, the obtained product (functionalized cork, C_f_) was purified by filtered under vacuum washing with DMF and dried. Then, 3.25 g of C_f_ was dispersed in 195 mL of toluene by magnetic stirring in a round flask and 42.7 mL of *n*BA (1.5 M) was slowly added to the mixture. Then, the flask was closed with a septum and nitrogen was bubbled into the mixture. Finally, 0.192 g of AIBN (0.006 M) was added and the mixture was heated up to 70 °C to start the polymerization. The reaction was stopped after 24 h, and the product (C_m_) was washed with toluene, isopropanol, and water to remove any excess of the unreacted monomer. After this, the product was filtered and dried under vacuum. Characterization of C, C_f_, and C_m_ was done by infrared spectroscopy via attenuated total reflectance (ATR) in a Bruker Alpha spectrometer. The morphology of the particles was examined by scanning electron microscopy (SEM) in an FEI Nova NanoSEM 450 microscope equipped with a field-emission gun. The particles were previously sputtered with a few nm layer of Au in a Balzers SCD 004 Sputter Coater.

### Composite Fabrication for Additive Manufacturing

**Figure 1:**
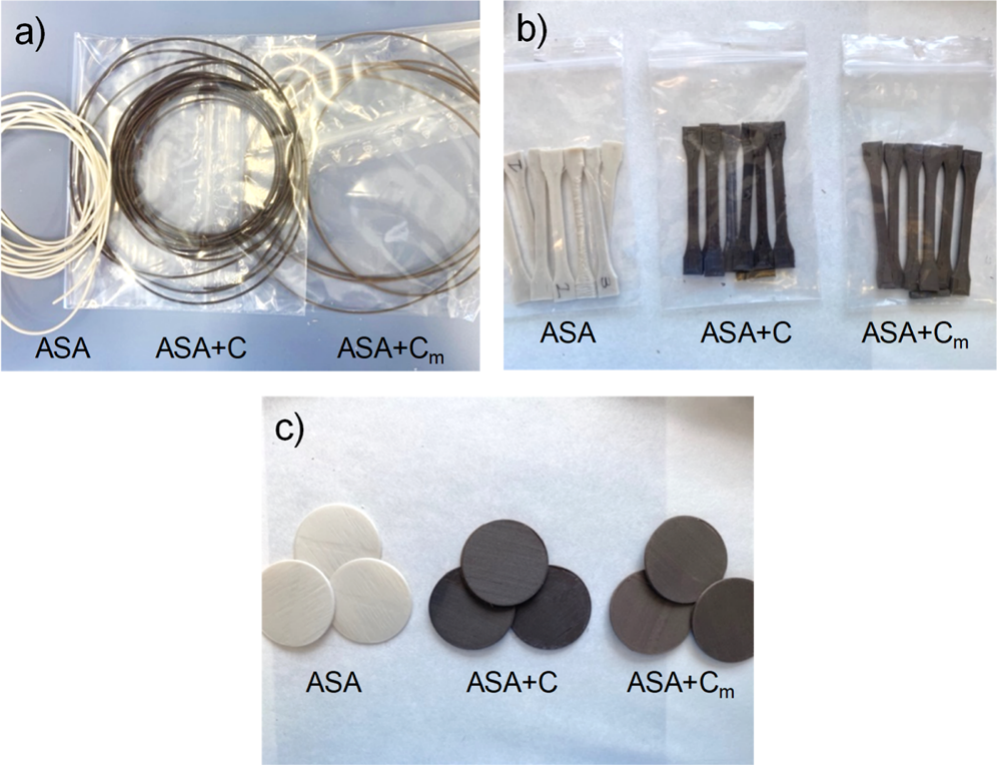
(a) Extruded filaments, (b) 3D-printed tensile testing specimens, and (c) 3D-printed thermal conductivity specimens of ASA, ASA + C, and ASA + C_m_.

**Scheme 1:**
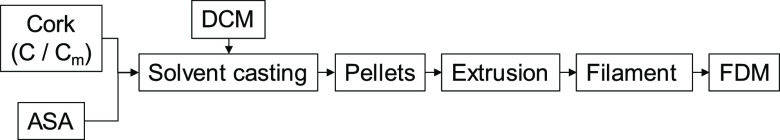
Manufacturing Process of the Cork-Based Composites

The fabrication of a cork-based composite filament valid for FDM is carried out as depicted in Scheme 1. First, 200 mL of DCM was added to 50 g of ASA. When the polymer was dissolved forming a viscous paste, 2.5 g of cork powder (either C or C_m_) was added and dispersed into the solution. The blend was mechanically stirred to ensure the formation of a homogeneous composite with 5 wt % of cork. Depending on the cork particles used, these composites are labeled as ASA + C or ASA + C_m_. The mixture was placed on aluminum trays, and the solvent was removed by heating at 60 °C for at least 24 h. Then, the films formed were cut into pellets of approximately 3 mm in size. These pellets were introduced afterward in an oven at 60 °C for at least 3 h prior to extrusion to remove any moist traces in the material. Then, the pellets were extruded in a Noztek single-screw extruder (26:1 L/D) at 60 rpm and 260 °C and a filament of ca. 1.75 mm was obtained. This process was also done with pure ASA in the absence of cork as a control. Produced filaments are shown in (Figure 1a).

### Additive Manufacturing of Cork Composites

CAD files in the .stl format of 1BA tensile testing specimens and discs of a 5 mm thickness and a 50 mm diameter for thermal conductivity testing according to ASTM D638 and ASTM E1530 standards, respectively, were loaded in the IdeaMaker 4.0.1 software, and a Gcode file was created with the printing conditions. Tensile testing specimens were designed with a core–shell infill pattern, while the discs for thermal conductivity testing were designed following a crisscross infill pattern. An infill of 100% was used in both cases. The printing temperature and speed were set to 245 °C and 50 mm/s, respectively. The platform temperature was set to 100 °C in order to ensure a good adhesion of the first layer of the material. The rest of the printing conditions were left by default. The specimens were then printed in a Raise 3D Pro2 FDM printer equipped with a 0.4 mm diameter nozzle using the previously extruded ASA, ASA + C, and ASA + C_m_ filaments. (Figure 1b,c) shows the 3D-printed specimens fabricated with the different materials by FDM.

### Cork Composite Characterization

Tensile testing of the printed specimens was performed using a universal testing machine (Shimadzu) at a constant speed of 1 mm/min according to ASTM D638. At least five specimens were tested for each material. The density of the materials was measured by weighing extruded filaments of ca. 30 cm length and 3D-printed objects with a known volume. The results were compared with the theoretical calculations by applying the rule of mixtures using the density values of ASA and cork given by the suppliers. Young’s modulus (*E*), tensile strength (σ_max_), elongation at break, specific modulus (*E*/ρ), and specific tensile strength (σ_max_/ρ) values were dissected for each one of the measured specimens. The results were averaged, and standard deviations were presented as error bars. Analysis of variance (ANOVA) with a significance level of α = 0.05 and Tukey's test were performed to determine if there were statistically significant differences between the results. SEM analyses were carried out using an FEI Nova NanoSEM 450 microscope equipped with a field-emission gun. Samples were previously sputtered with a few nm layer of Au in a Balzers SCD 004 Sputter Coater. ImageJ software was used to calculate the size of the cork particles. The thermal conductivity measurements were done in a TA Instruments DTC-25 conductivity meter according to the ASTM E1530 standard. At least three different samples were measured for each material. The results were averaged, and the standard deviations were presented as error bars.

## Results and Discussion

The cork powder received was first sieved, and the fraction with a particle size between 63 and 125 μm was taken. This fraction was washed and dried prior to any further use. Before performing the surface modification of the cork particles to enhance the compatibility with the polymer matrix, the amount of hydroxyl groups in the surface is quantified. Cork is composed mainly of suberin, lignin, and cellulose. These macromolecules are rich in hydroxyl groups.^5^ For this purpose, these functional groups are acetylated with an excess of acetic anhydride under similar conditions as described in the ASTM D1957 standard. It is important to notice that this is not the total value of hydroxyl groups present in the bulk structure of cork but only the ones present in the surface of the particles, which are available for surface modification reactions. The excess of acetic anhydride is then hydrolyzed with water, and the amount of acetic acid obtained is titrated with a 0.5 M NaOH solution. A control in the absence of cork allows to determine the total amount of acetic acid that can be formed, and the difference between these values allows to quantify the OHN. This process is repeated three times, and the OHN of the cork particles is given as the mmol of hydroxyl groups in the surface per gram of cork. Table 1 shows the OHN obtained after these assays. Hence, it was determined that the amount of hydroxyl groups available to be functionalized is 0.80 ± 0.37 mmol −OH/g_cork_. A detailed explanation of the procedure is given in the Supporting Information.

**Table 1:** OHN Values for Three Independent Measurements

#repeat	OHN (mmol −OH/g_cork_)
1	0.451
2	1.202
3	0.742

**Figure 2:**
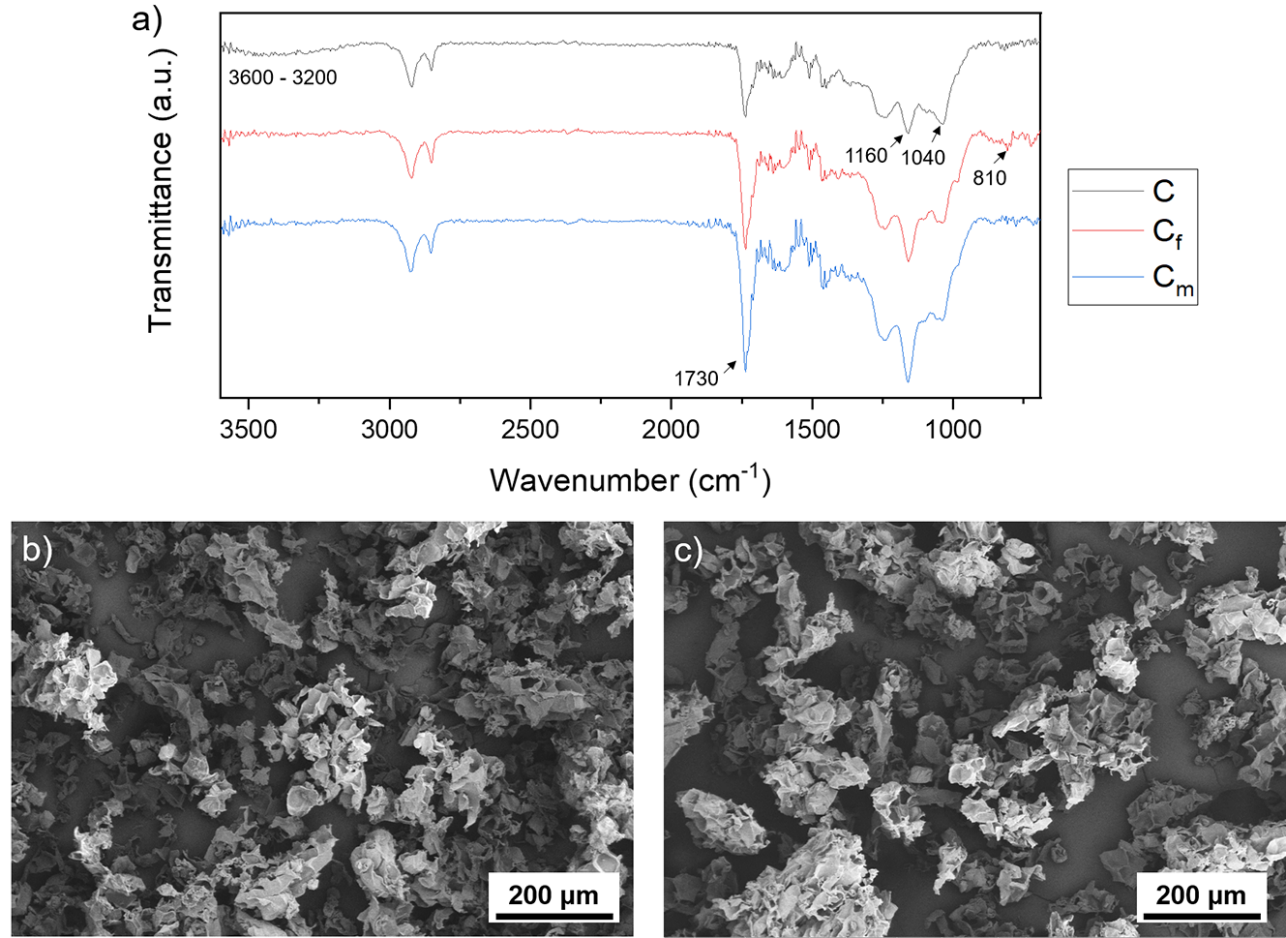
(a) ATR spectra of C, C_f_, and C_m_. SEM micrographs of (b) C and (c) C_m_.

**Scheme 2:**
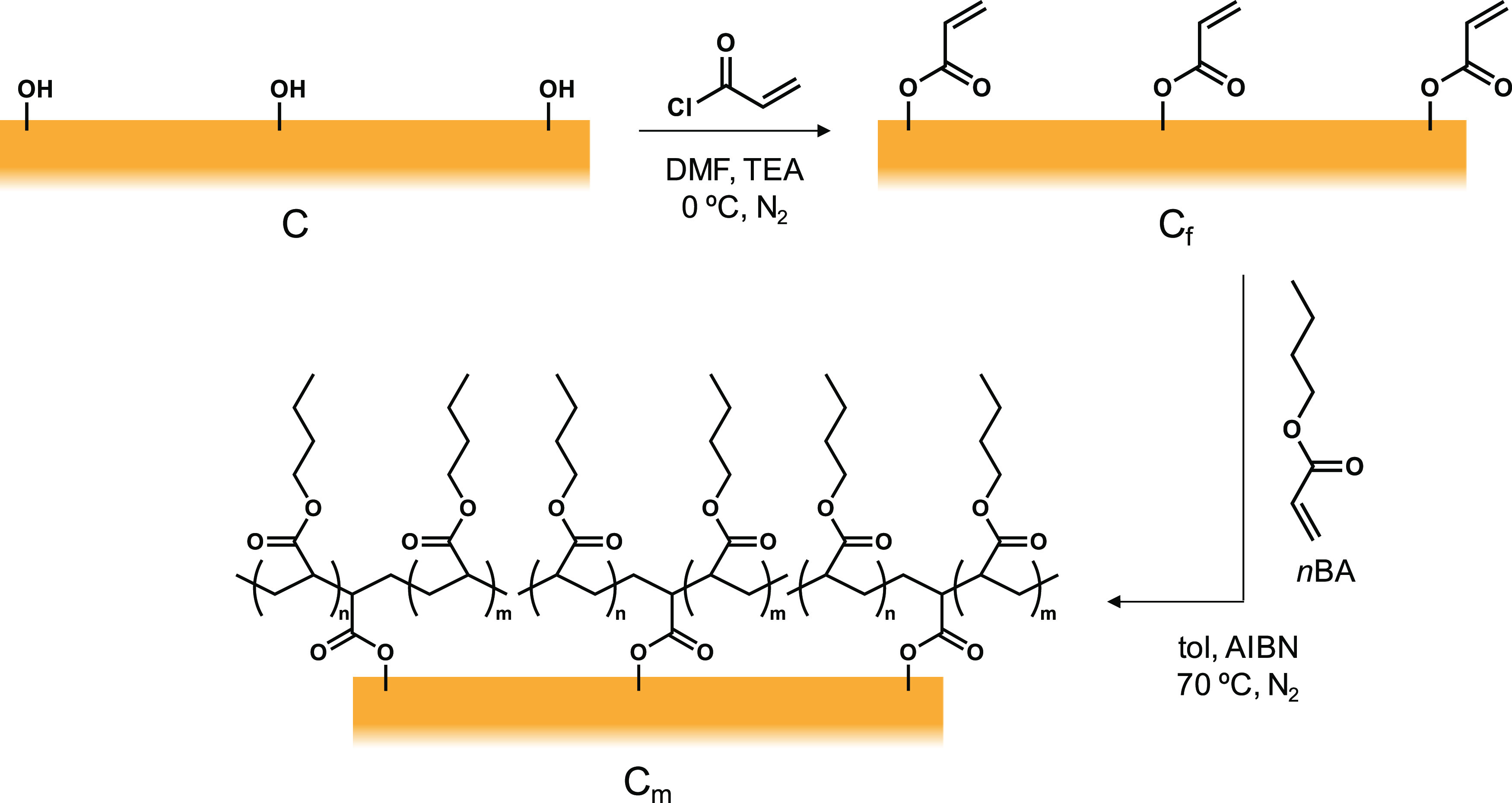
Surface Polymerization of the Cork Particles The first reaction consists of the esterification of the hydroxyl groups on the surface with acryloyl chloride to obtain C_f_. The second reaction is the polymerization of *n*BA into PBA. Part of the PBA synthesis is expected to happen in the surface of C_f_ due to the presence of polymerizable vinyl groups, obtaining C_m_.

Once the amount of hydroxyl groups available for functionalization is known, the cork particles are chemically modified. Scheme 2 depicts the procedure followed to obtain C_m_ in a two-step approach. First, C is treated with an excess of acryloyl chloride to have polymerizable moieties in the surface of the cork particles than can further participate in a subsequent polymerization reaction. After reaction and purification of the cork, C_f_ is obtained. This functionalized compound is then used as a co-monomer in a typical radical polymerization reaction using *n*BA as a monomer and AIBN as an initiator in order to create a polymeric layer of PBA in the surface of the cork particles. This is expected to significantly enhance the compatibility of the cork with the ASA matrix since PBA is one of the polymers present in this terpolymer. Figure 2a shows the infrared spectra of the cork particles before and after the functionalization and polymerization. A small, broad band of low intensity is observed at 3200–3600 cm^–1^, corresponding to the O–H stretching of C. This band disappears for C_f_ and C_m_, indicating the reaction of the hydroxyl moieties in the surface of the cork particles, as depicted in Scheme 2. It is also interesting to compare the relative intensity of the C–O of acrylates at 1160 cm^–1^ with that of polysaccharides (i.e., cellulose and hemicellulose, naturally present in cork) at 1040 cm^–1^. These signals have similar intensity for C. However, the peak at 1160 cm^–1^ increases gradually in a significant manner for C_f_ and C_m_, while the peak at 1040 cm^–1^ does not vary its intensity. A similar trend can be observed for the C=O stretching at 1730 cm^–1^ for C_f_ and C_m_, indicating the successful immobilization of acryloyl chloride and PBA, respectively, in the surface of the cork particles.^7,29^ Moreover, a small peak at 810 cm^–1^ corresponding to the vinyl group of *n*BA can be observed for C_f_, indicating that the monomer is immobilized in the surface of the cork particles after the first reaction. This signal disappears for C_m_, indicating that the polymerization and purification were carried out adequately.^31^ Further proof of the successful modification of C was the highly hydrophobic behavior of C_m_, which floated rapidly to the surface when immersed in water. This effect was not observed for C particles (Figure S1). The morphology of the particles was also observed before and after the surface polymerization. Figure 2b,c shows that there are no significant differences in the structure of the cork particles after the chemical modification, evidencing that the morphology of the cork remains unaltered, at least on a microscale. The average size of the particles was quantified as 84 ± 58 and 91 ± 64 μm for cork particles before and after modification, respectively. These differences are not significant and are in good agreement with the expected theoretical cork sizes after sieving.

Then, the composites containing 5 wt % of cork (either C or C_m_) were manufactured via solvent casting to obtain ASA + C and ASA + C_m_. This strategy does not require the use of high temperatures during the compounding process, as in twin screw extrusion, avoiding the degradation of the cork particles. A control was also done with ASA only by solvent casting for comparative purposes. The pellets obtained are well dried under vacuum and at 60 °C to ensure that all the traces of DCM occluded and moist are removed. The ASA, ASA + C, and ASA + C_m_ pellets were then extruded at 260 °C to obtain a filament with a homogeneous diameter of 1.70 ± 0.5 mm, suitable for FDM. The filaments produced also showed a very uniform color, implying that the cork particles are homogeneously distributed in the composite material. Interestingly, ASA + C filaments exhibit a darker color than ASA + C_m_, which does not vary after the specimens are printed at 245 °C by FDM, as depicted in Figure 1. The color changes in the cork at temperatures in the range of 200–350 °C result from decomposition of hemicellulose and low-molecular-weight lignin and suberin to a lesser extent.^27,32^ However, previous reports indicate that irreversible thermal degradation of cork in an air atmosphere starts at 258 °C, evidencing that cork powder can be processed at temperatures below 250 °C without significant changes in the chemical composition, even if there are color changes.^7,33^ In our case, the extrusion temperature was slightly higher, 260 °C, since lower temperatures caused clogging in the screw and made it difficult to form a suitable filament for FDM. However, given the low residence time of the composite inside the extruder (less than 1 min), it was assumed that the degradation in this step was practically negligible. The color differences between ASA + C and ASA + C_m_ seem to indicate that the PBA coating layer on the C_m_ particles acts as a protective barrier that prevents, or at least delays, the degradation of the particles.

**Figure 3:**
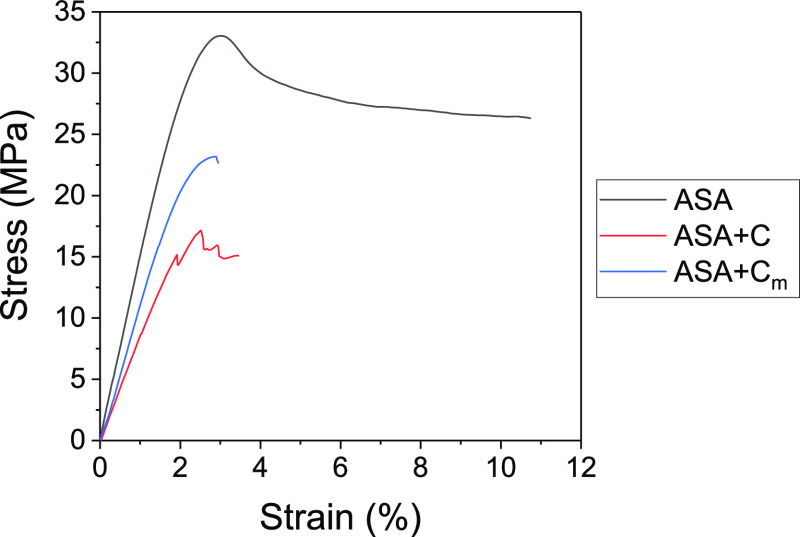
Representative tensile testing curves for ASA (black), ASA + C (red), and ASA + C_m_ (blue).

The mechanical properties of the printed materials were studied by tensile testing. Stress–strain curves of ASA, ASA + C, and ASA + C_m_ are presented in Figure 3. The ASA curve shows that the material is first stretched elastically until it reaches a maximum value around 3% strain and then undergoes a certain plastic deformation before failure. On the other hand, the ASA + C curve shows a very brittle material without plastic deformation. An interesting series of sudden jumps can be observed prior to failure caused by delamination of the specimen due to partial fracture of the printed layers. This behavior is characteristic of a poorly printed composite, showing no compatibility between the matrix and the filler. ASA + C_m_ curve shows an intermediate behavior between ASA and ASA + C_m_, indicating that the surface polymerization of PBA on the cork particles acts effectively as a compatibilizing agent, contributing to enhance the mechanical properties of the cork composite.

**Figure 4:**
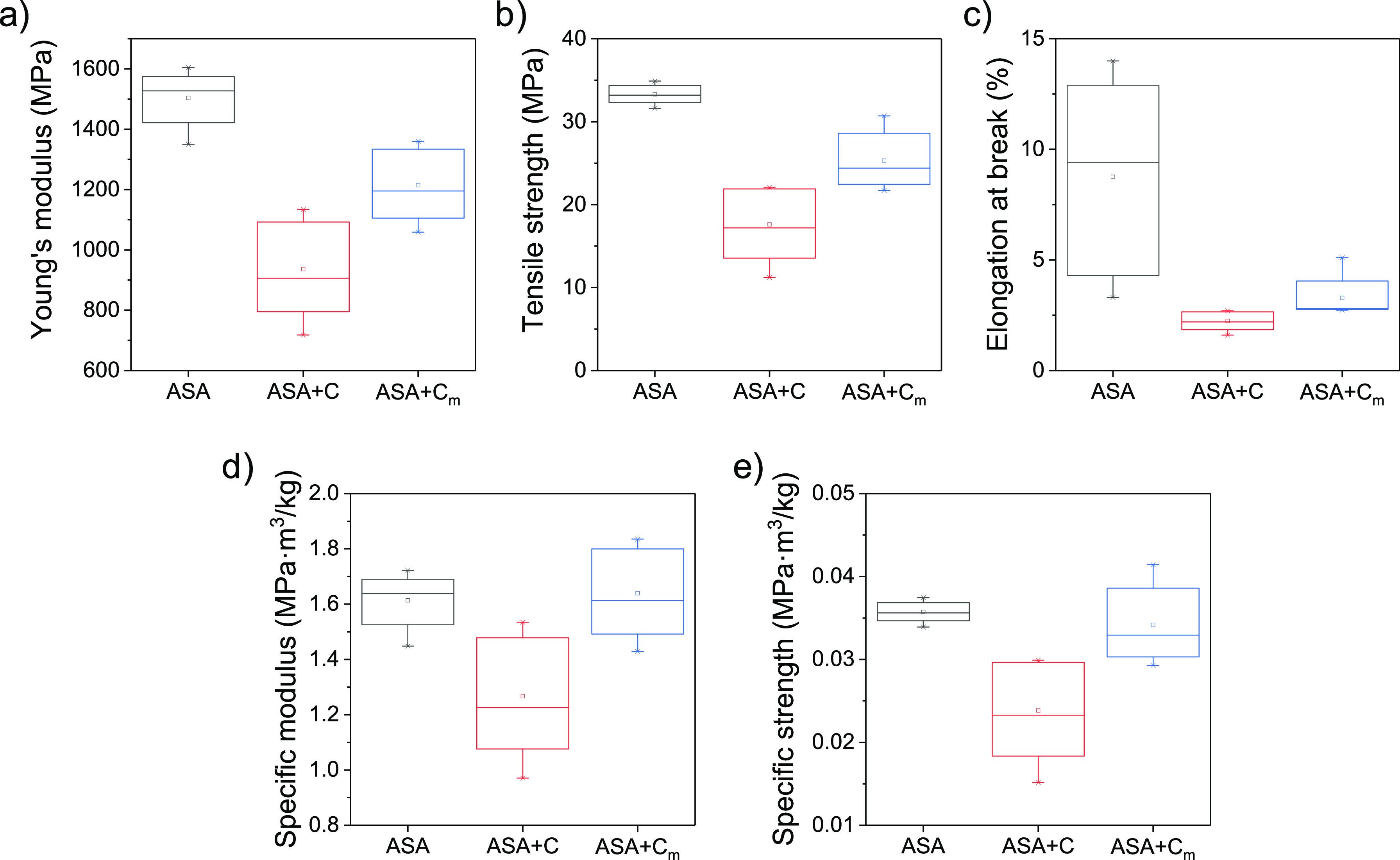
Box plots of (a) Young’s modulus, (b) tensile strength, (c) elongation at break, (d) specific modulus, and (e) specific tensile strength dissected from five independent measurements for ASA, ASA + C, and ASA + C_m_.

A summary of the mechanical properties dissected from these curves (Young’s modulus, tensile strength, and elongation at break), including statistical analysis, are summarized in the form of box plots in (Figure 4a–c). These results show that the surface modification of cork contributed to enhance the mechanical properties of the composites since ASA + C_m_ exhibits a statistically higher Young’s modulus and tensile strength than ASA + C. However, neither ASA + C nor ASA + C_m_ present better mechanical properties than ASA, as expected.

In fact, cork cannot be used as a reinforcing agent since its mechanical properties are lower than those of ASA. Similar results were previously reported for other cork composites based on PLA,^27^ PP, or PE.^34^ All these studies conclude that the use of compatibilizing agents contributes to improve the adhesion of cork to the polymeric matrix. However, due to the properties of the cork, the mechanical performance of the composite generally decreases when compared to the unmodified polymer.^7^ The addition of cork may increase the strength of the material only if the original polymeric matrix used is rather weak, as reported for PMMA composites, which undergo an increase of the tensile strength from 3 to 6 MPa, approximately.^30^ When the cork content is fixed, the enhancement of the cork-matrix compatibility can increase the mechanical properties up to 40% when compared to composites containing unmodified cork.^34^

A more detailed analysis of the mechanical properties of ASA, ASA + C, and ASA + C_m_ was done by applying the rule of mixtures using the density values provided by the manufacturers. In particular, the upper and lower bounds of the Young’s modulus were predicted for ASA composites with cork particles (see the Supporting Information for more details). It was found that ASA + C_m_ exhibits practically the maximum Young’s modulus predicted by the rule of mixtures, which indicates a good adhesion between C_m_ and ASA. On the other hand, the cork particles may be added to lighten the weight of the material. In this regard, it is also interesting to compare the specific properties (defined by the ratio between the measured property and the density of the composite) of the materials, which are presented in Figure 4d,e. For this purpose, the density of the 3D-printed specimens was experimentally calculated (see Table S2). Statistical analysis via ANOVA and Tukey’s tests indicate that the specific modulus (*E*/ρ) and specific strength (σ_max_/ρ) values of ASA + C_m_ are similar (i.e., not significantly different) to those of ASA. However, these specific properties are statistically lower for ASA + C. This evidences that ASA + C_m_ is a competitive material in the design of light, stiff, and strong beams or ties for applications where a minimum structural weight is required.

**Figure 5:**
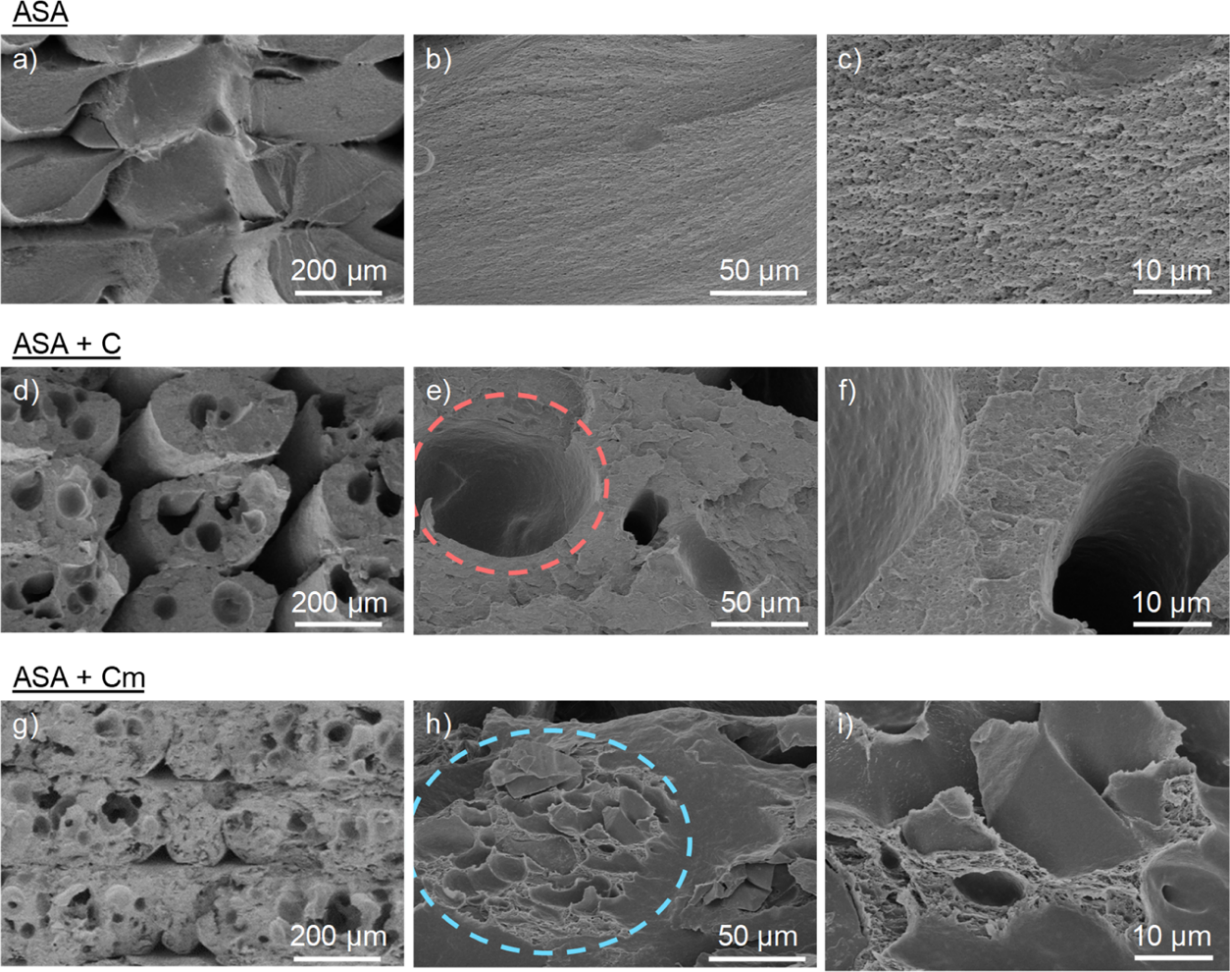
Fracture surface of the tensile testing specimens of (a–c) ASA, (d–f) ASA + C, and (g–i) ASA + C_m_. The dashed circle in (e) shows the gap of a C particle that likely has come off the surface after tensile testing, while the dashed circle in (h) shows a C_m_ particle well embedded in the ASA matrix.

SEM analyses of the fracture surface of the composites were done to obtain more information about the structural behavior of these materials. Figure 5a–c shows the transversal section of ASA composites, where some gaps between layers and roads, characteristic of FDM manufacturing, can be seen at low magnifications. The fracture observed is rather clean and the surface is relatively smooth, even though some minor roughness can be observed at higher magnifications, which could be attributed to the plastic deformation of the material before breaking. The fracture surface observed in ASA + C (Figure 5d–f) shows the highest porosity of the three materials studied. This porosity is probably originated from two different factors: first, the interlayer porosity, higher than that in the case of pure ASA, caused by a poor flow of the deposited materials during the FDM process. The presence of cork particles increases the viscosity of the molten material,^26^ which provokes a higher interlayer porosity when printing. Second, several pores are observed within the roads of the deposited material, likely due to the presence of cork. Although the cork particles are not observed, the gaps found in the fracture surface match the size of the cork particles (see, for instance, the dashed circle in Figure 5e). Thus, it is probable that the cork particles may have come off during or after material failure, which supports the hypothesis that the compatibility between ASA and cork is very poor. The high porosity is also responsible of the decrease of the mechanical properties of ASA + C presented in Figure 4. ASA + C_m_ composites present an interesting fracture surface, as shown in Figure 5g–i. The interlayer porosity observed at low magnifications is smaller when compared to ASA + C, which could be expected since in this case, the material flowed adequately during the printing process. As previously discussed, this is likely due to the PBA polymeric layer on the surface of the cork particles that acts as a plasticizer, decreasing the viscosity of the molten material. The porosity within the roads of the deposited material appears to be homogeneously distributed along the fracture surface. When these pores are observed at higher magnifications, the characteristic cellular structure of the cork particles can be appreciated, well embedded by the ASA polymeric matrix (see the dashed circle in Figure 5h). This evidences that C_m_ remains in the surface after fracture, maintaining its structural properties, contrary to what was observed in the case of ASA + C.

**Figure 6:**
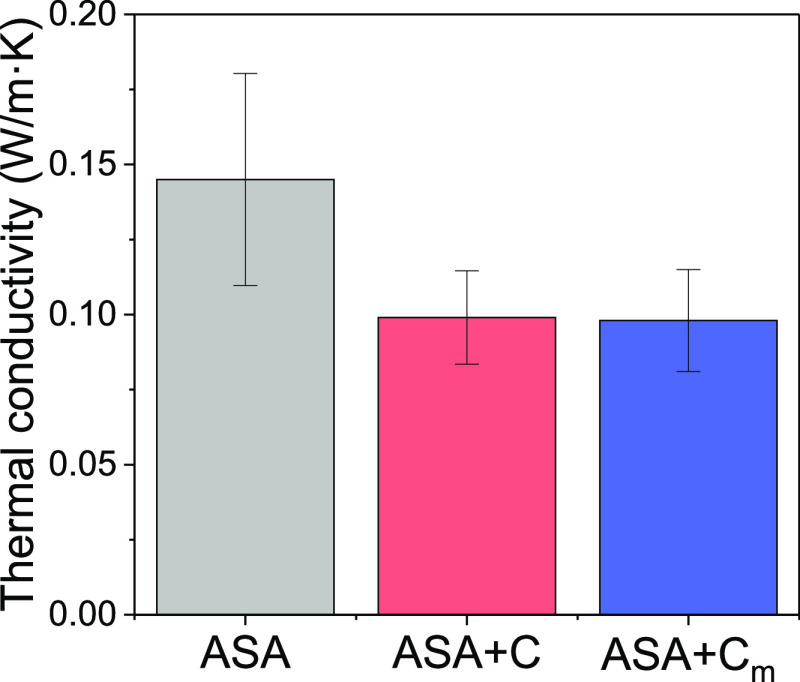
Thermal conductivity values of ASA, ASA + C, and ASA + C_m_.

Finally, the thermal conductivity of the composites was studied. Due to its cellular morphology, cork has a very low thermal conductivity (0.045 W/m K), close to that of air, which makes it an ideal candidate as a thermal insulator. Figure 6 shows the thermal conductivity of ASA, ASA + C, and ASA + C_m_, which remarkably decreases from (0.145 ± 0.035) W/m K for pure ASA to (0.099 ± 0.015) W/m K and (0.098 ± 0.017) W/m K for ASA + C and ASA + C_m_, respectively. These values are slightly below those theoretically calculated for these composites, where a thermal conductivity of 0.11 ± 0.01 W/m K is predicted for ASA composites containing 5 wt % cork, according to the rule of mixtures. Different factors may have contributed to this. On one hand, the interlayer porosity, characteristic of the FDM process, has created air bubbles inside the printed materials, which is expected to enhance their insulating behavior. This is supported by the density results measured experimentally for the 3D-printed objects, which are lower than the theoretical ones (see Table S2). On the other hand, the low density values, together with the SEM results [see (Figure 5h)], also indicate that the microstructure of the cork particles is not altered and there is no densification of the cork particles during manufacturing of the composites, which would lessen their insulating behavior. Hence, these composites contain a high amount of air, either in the form of interlayer porosity or trapped within the cellular walls of cork, making these materials good candidates as insulators.

## Conclusions

This research evidences the possibility of using cork particles (received as an agro-residue from local industries) as an additive in the development of composites suitable for their manufacturing via FDM. The composites containing 5 wt % (22.54 vol%) of cork particles (either C or C_m_) were manufactured by solvent casting at room temperature and subsequent extrusion in a single-screw extruder with a short barrel, which minimizes the processing of cork at high temperatures (i.e., above 250 °C). This strategy is an alternative to composite compounding via twin-screw extrusion, where longer residence times are typically required and the cork particles could be degraded to a higher extent. The densities of the filaments and objects manufactured were similar or lower than those predicted theoretically, suggesting that there is no densification of the cork particles during the manufacturing process. Furthermore, the strategy proposed for the modification of the cork by surface polymerization of PBA greatly enhanced its compatibility with the ASA matrix as it was observed by SEM in the aspect of the fracture surface and in the increase of the mechanical properties of ASA + C_m_ when compared to ASA + C. ASA + C_m_ exhibited similar specific modulus and specific strength values to pure ASA, indicating that it is a good candidate for stiff and strong beams and ties, where lightweight materials are required. ASA + C_m_ composites showed significantly lower thermal conductivity than ASA due to the air trapped as interlayer porosity and in the cellular structure of the cork. Thus, the combination of these properties makes ASA + C_m_ a good candidate as a lightweight insulating material, allowing to valorize a residue and contributing to the circular economy.

## Supporting Information Available

Calculation of the OHN of the cork particles, correlation of the experimental mechanical properties with the rule of mixtures, and density of materials (PDF)

## Notes

Data supporting the findings of this study are available from the corresponding author (A.S.d.L.) upon reasonable request.
