# Supplementary material for: Polymer Composites with Cork Particles Functionalized by Surface Polymerization for Fused Deposition Modeling
Source: ACS Appl Polym Mater. 2022 Jan 13;4(2):1225–33. doi: 10.1021/acsapm.1c01632 (PMC8848549; doi:10.1021/acsapm.1c01632)
Supplement: Supplementary file 1 — ap1c01632_si_001.pdf [file ap1c01632_si_001.pdf]

Polymer composites with cork particles functionalized by surface polymerization for fused deposition modelling

SUPPORTING INFORMATION

Alberto S. de León*, Fernando Núñez-Gálvez, Daniel Moreno-Sánchez,
Natalia Fernández-Delgado, Sergio I. Molina

Dpto. Ciencia de los Materiales, I. M. y Q. I., IMEYMAT, Facultad de Ciencias, Universidad de Cádiz, Campus Río San Pedro, s/n, 11510 Puerto Real (Cádiz), Spain.

*corresponding author: alberto.sanzdeleon@uca.es

Keywords: cork, composites, additive manufacturing, fused deposition modelling, surface modification, circular economy, renewable resources.

Calculation of the hydroxyl number (OHN) of the cork particles

The procedure to obtain the OHN of the cork particles (i.e. the amount of –OH groups available for reaction) is adapted from ASTM D1857 as follows:
First, the acetic anhydride $(CH_3CO)_2O$ reacts with the hydroxyl groups of the cork particles $R\text{-}OH$ producing the acetylation of the cork particles and acetic acid CH_3COOH as shown in reaction (1):

$$R\text{-}OH + (CH_3CO)_2O \rightarrow R\text{-}OOC\text{-}CH_3 + CH_3COOH \quad (1)$$

In these conditions, it can be ensured that all the $R\text{-}OH$ moieties of the cork particles will be acetylated, since $(CH_3CO)_2O$ is in excess and therefore the amount of acetylated groups is equivalent to the OHN. Then, the excess of $(CH_3CO)_2O$ is hydrolyzed with H_2O according to reaction (2):

$$(CH_3CO)_2O + H_2O \rightarrow 2\,CH_3COOH \quad (2)$$

Finally, all the acetic acid CH_3COOH produced in (1) and (2) is titrated with a 0.5 M NaOH solution, as indicated in reaction (3):

$$CH_3COOH + NaOH \rightarrow CH_3COONa + H_2O \quad (3)$$

In the control experiment (i.e. in absence of cork), (1) does not take place, therefore all the CH_3COOH is produced according to (2). Reaction (1) produces 1 mol $CH_3COOH/(CH_3CO)_2O$ while reaction (2) produces 2 mol $CH_3COOH/(CH_3CO)_2O$. The OHN is proportional to CH_3COOH produced in (1), therefore to the difference between the $NaOH$ needed after (1) and (2) in presence of cork $V_{NaOH,cork}(mL)$ and the control $V_{NaOH,control}$. Precisely, the OHN can be calculated as:

$$\frac{mmol\text{ -}OH}{g_{cork}} = \frac{\left(V_{NaOH,control}(mL) - V_{NaOH,cork}(mL)\right) \cdot 0.5\; mmol\; NaOH/mL}{m_{cork}\;(g)}$$

The experimental results for the three repeats done are presented in **Table S1**.

Table S1. Amount of cork used (m_{cork}), NaOH volume needed for titration after (1) and (2) in presence of cork ($V_{NaOH,cork}$), NaOH volume needed for the control ($V_{NaOH,control}$) and OHN (mmol –OH/g_{cork}) for each repeat.

**#repeat**	**m_{cork} (g)**	**$V_{NaOH,cork}$ (mL)**	**$V_{NaOH,control}$ (mL)**	**OHN (mmol -OH/g_{cork})**
1	0.3325	9.7	10.0	0.451
2	0.3327	8.4	9.2	1.202
3	0.3366	8.3	8.8	0.742

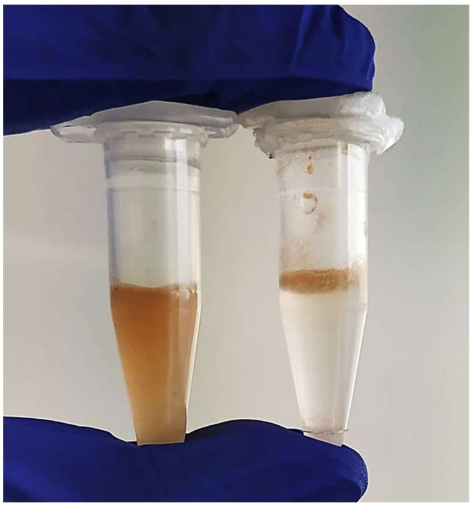

Figure S1. Eppendorf tubes containing aqueous solutions of approximately 5 mg/mL of C (left) and C_m (right), seconds after being shaken vigorously. It can be observed that C particles remain dispersed in water, while C_m rapidly float towards the surface due to their highly hydrophobic behavior.

Correlation of the experimental mechanical properties with rule of mixtures

The rule of mixtures states that the upper and lower bounds for the Young's modulus of a composite made of a polymeric matrix and equiaxed particles can be estimated as:

$$E_c(upper) = E_m y_m + E_p y_p$$

$$E_c(lower) = \frac{E_m E_p}{y_m E_p + y_p E_m}$$

Where E is the Young's modulus, y the volume fraction and sub-indexes c, m and p represent the composite, matrix (ASA) and particles (cork), respectively.

Taking as a reference E_m = 1700 MPa (from experimental tensile testing of 3D printed pure ASA specimens, see **Table 2**) and E_p = 31.5 MPa[1], the graph for the rule of mixtures of the Young's modulus of the system ASA + cork is shown in **Figure S2**. In this figure there are also included the experimental values dissected from the tensile testing curves of ASA+C and ASA+Cm. It must be noted that the volume content for these composites containing 5 wt% cork corresponds to 22.5 vol% due to the low density of cork (200 kg/m^3). The graph clearly shows how the composites containing C_m increase the Young's modulus up to practically the upper limit of the theoretical calculations, evidencing that the surface polymerization of cork with PBA greatly enhances the compatibility between the particles and the ASA matrix.

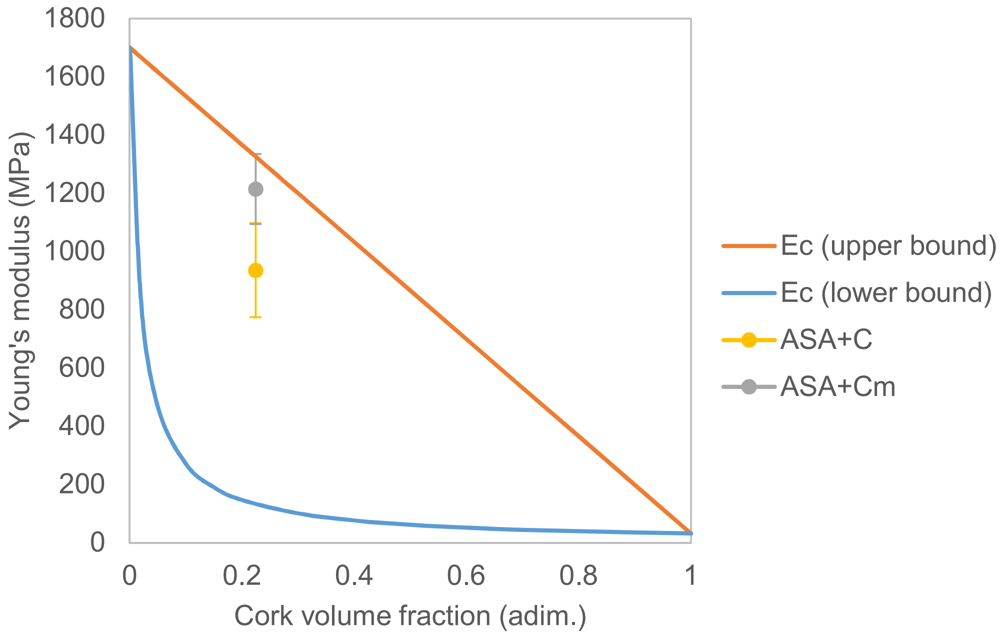

Figure S2. Predicted upper (orange line) and lower (blue line) bounds of ASA + cork composites according to the rule of mixtures. The experimental values obtained for the Young's modulus of ASA+C and ASA+Cm are presented in yellow and grey respectively.

(1) Ansys Granta EduPack software, ANSYS, Inc., Cambridge, UK, **2019**.

Table S2. Density values of ASA, ASA+C and ASA+C_m, calculated theoretically from the values provided by the suppliers and measured experimentally from the filaments and 3D-printed objects. Experimental values of the extruded filament are in well-agreement with the theoretical ones, indicating that the filament was successfully printed, without significant porosity or densification of the cork particles. Experimental values for the 3D-printed objects suggest that there is a certain interlayer porosity (around 15%), which causes a decrease in the density of the objects. These results are in well-agreement with the SEM images.

	**Density (kg/m^3)**		
	**Theoretical**	**Extruded filament**	**3D-printed objects**
**ASA**	1100	1108 ± 83	932 ± 43
**ASA+C**	898	861 ± 63	740 ± 47
**ASA+C_m**	898	881 ± 18	742 ± 69
